# Multimodality Imaging in Cranial Giant Cell Arteritis: First Experience with High-Resolution T1-Weighted 3D Black Blood without Contrast Enhancement Magnetic Resonance Imaging

**DOI:** 10.3390/diagnostics14010081

**Published:** 2023-12-29

**Authors:** Jane Maestri Brittain, Michael Stormly Hansen, Jonathan Frederik Carlsen, Andreas Hjelm Brandt, Lene Terslev, Mads Radmer Jensen, Ulrich Lindberg, Henrik Bo Wiberg Larsson, Steffen Heegaard, Uffe Møller Døhn, Oliver Niels Klefter, Anne Katrine Wiencke, Yousif Subhi, Steffen Hamann, Bryan Haddock

**Affiliations:** 1 Department of Clinical Physiology and Nuclear Medicine, Rigshospitalet, DK-2100 Copenhagen, Denmark; bryan.haddock@regionh.dk; 2 Department of Ophthalmology, Rigshospitalet, DK-2600 Glostrup, Denmark; michael.stormly.hansen@regionh.dk (M.S.H.); steffen.heegaard@regionh.dk (S.H.); oliver.niels.klefter.01@regionh.dk (O.N.K.); anne.katrine.wiencke@regionh.dk (A.K.W.); ysubhi@gmail.com (Y.S.); steffen.ellitsgaard.hamann@regionh.dk (S.H.); 3 Department of Radiology, Rigshospitalet, DK-2100 Copenhagen, Denmark; jonathan.frederik.carlsen@regionh.dk (J.F.C.); andreas.hjelm.brandt.02@regionh.dk (A.H.B.); 4 Department of Clinical Medicine, University of Copenhagen, DK-2200 Copenhagen, Denmark; lene.terslev.01@regionh.dk; 5 Department of Rheumatology and Spine Diseases, Rigshospitalet, DK-2600 Glostrup, Denmark; uffe.doehn@regionh.dk; 6 Department of Clinical Physiology and Nuclear Medicine, Bispebjerg Hospital, DK-2400 Copenhagen, Denmark; mads.radmer.jensen@regionh.dk; 7 Functional Imaging Unit, Department of Clinical Physiology and Nuclear Medicine, Rigshospitalet, DK-2600 Glostrup, Denmark; ulrich.lindberg@regionh.dk (U.L.); henrik.bo.wiberg.larsson@regionh.dk (H.B.W.L.); 8 Eye Pathology Section, Department of Pathology, Rigshospitalet, DK-2100 Copenhagen, Denmark; 9 Department of Clinical Research, University of Southern Denmark, DK-5000 Odense, Denmark; 10 Department of Ophthalmology, Zealand University Hospital, DK-4000 Roskilde, Denmark

**Keywords:** giant cell arteritis, vascular ultrasound, FDG PET/CT, temporal artery biopsy, 3D black blood, MRI, non-contrast enhancement, multimodality imaging

## Abstract

In order to support or refute the clinical suspicion of cranial giant cell arteritis (GCA), a supplemental imaging modality is often required. High-resolution black blood Magnetic Resonance Imaging (BB MRI) techniques with contrast enhancement can visualize artery wall inflammation in GCA. We compared findings on BB MRI without contrast enhancement with findings on 2-deoxy-2-[^18^F]fluoro-D-glucose positron emission tomography/low-dose computed tomography (2-[^18^F]FDG PET/CT) in ten patients suspected of having GCA and in five control subjects who had a 2-[^18^F]FDG PET/CT performed as a routine control for malignant melanoma. BB MRI was consistent with 2-[^18^F]FDG PET/CT in 10 out of 10 cases in the group with suspected GCA. In four out of five cases in the control group, the BB MRI was consistent with 2-[^18^F]FDG PET/CT. In this small population, BB MRI without contrast enhancement shows promising performance in the diagnosis of GCA, and might be an applicable imaging modality in patients.

## 1. Introduction

Giant cell arteritis (GCA) is the most frequent primary chronic vasculitis affecting medium- and large-sized arteries, with the highest incidence among Northern Europeans ≥50 years of age [1,2,3]. Timely and accurate diagnosis of GCA is important to prevent potentially severe morbidity, including permanent vision loss and stroke. Classic symptoms including jaw claudication, new-onset headache, cutaneous scalp allodynia, and visual disturbances are not always present, and the clinical diagnosis of GCA can be challenging [4]. There are no clinical diagnostic criteria for GCA, and prompt initiation of high-dose steroid treatment is effectuated on clinical suspicion to reduce the risk of serious complications. Therefore, an early imaging test is needed to support the clinical suspicion of GCA. According to the recently published European League Against Rheumatism (EULAR) recommendations in imaging in cranial GCA, vascular ultrasound (US) is the first-line imaging of choice [5]. Hybrid imaging with 2-deoxy-2-[^18^F]fluoro-D-glucose positron emission tomography/low-dose computed tomography (2-[^18^F]FDG PET/CT) and high-resolution Magnetic Resonance Imaging (MRI) with contrast enhancement are recommended alternative non-invasive imaging modalities [5]. Using MRI, an evaluation of the vessel wall and lumen diameter can be performed from pre- and post-gadolinium enhancement images. Like thickening of the wall and narrowing of the lumen, post-gadolinium enhancement of the vessel wall is indicative of mural inflammation in GCA [6]. In recent years, however, supplemental high-resolution black blood MRI (BB MRI) techniques have been used to suppress the signal from the flowing blood, allowing a clear visualization and characterization of structures juxtaposed to the blood pool, i.e., the vessel wall or perivascular space [7,8]. The use of contrast enhancement to reveal mural inflammation in intra- and extracranial vasculitis, even with blood suppression, has been evaluated [9,10]. Literature on the diagnostic quality of BB MRI with contrast enhancement imaging in intracranial vasculopathies is abundant, but for cranial GCA alone it is sparser.

The aim of this explorative study was to evaluate the use of 3D high-resolution T1-weighted black blood MRI to diagnose GCA, using the result of 2-[^18^F]FDG PET/CT as the reference standard; this was supported by the final clinical diagnosis, including results from US and temporal artery biopsy (TAB).

## 2. Materials and Methods

A group of ten patients and five controls was included in this prospective study. Patients were recruited from an ongoing prospective study evaluating the optimal diagnostic strategy in GCA, including US, 2-[^18^F]FDG PET/CT, and TAB. Patients who were suspected of having GCA were referred by specialists in either ophthalmology or rheumatology. After referral, patients underwent a clinical and neuroophthalmological examination including funduscopy and optical coherence tomography. Bloodwork, including C-reactive protein (CRP) and erythrocyte sedimentation rate (ESR) analysis, was performed. Furthermore, the initiation date of high-dose steroid therapy was noted. The final diagnosis was made six months after inclusion, and was based on initial/persistent symptoms, clinical findings, and results of neuroophthalmological examination, US, 2-[^18^F]FDG PET/CT, TAB, and blood work. Ten of these patients, suspected of GCA, had a supplemental BB MRI performed. Furthermore, five control subjects, who had a 2-[^18^F]FDG PET/CT performed as part of routine control for malignant melanoma, were recruited, and had a BB MRI performed. Inclusion criterion was that there were no pathological findings in the head region on the 2-[^18^F]FDG PET/CT scan. Exclusion criteria were ferromagnetic implants and severe claustrophobia. No other examinations of the control subjects were performed. Informed written consent was obtained from all patients and control subjects. The study was conducted according to the Declaration of Helsinki, and was approved by our local Research and Quality Improvement Board, Rigshospitalet (protocol 456_21) and the Legal Department of Rigshospitalet (project number 21016166).

### 2.1. Vascular US and Analysis

A GE Logiq^®^ E10 (Milwaukee, WI, USA) ultrasound scanner with 6–24 MHz Hockey-stick linear array transducers was used for all US scans. PRF, Doppler frequency, and Doppler gain were adjusted individually, for optimal flow depiction. The patients were examined in the supine position by an experienced rheumatologist. The temporal, facial and carotid arteries were examined bilaterally for hypoechoic wall thickening (the halo sign) in both the long and short axis. The temporal and facial arteries were also examined for presence of the compression sign (an incompressible artery, when pressure was administered with the transducer), as described elsewhere [11]. The presence of either halo or compression sign in one or more arteries was interpreted as a positive US sign for GCA as defined by the Outcome Measures in Rheumatology (OMERACT) US Large Vessel Vasculitis Group [12]. The US examination time was approximately 20–45 min.

### 2.2. PET/CT Image Acquisition and Analysis

After 4 h of fasting and confirmation that blood glucose level did not exceed 7 mmol/L, a dose of 3 MBq kg^−1^ FDG (max 500 MBq) was administrated intravenously. After one hour of resting, post injection, patients were scanned using a Siemens Biograph 64 PET/CT system (Siemens Healthcare, Erlangen, Germany). A conventional whole-body 2-[^18^F]FDG PET/CT scan, including the head and arms positioned along the truncus, was then performed, with 3 min per bed position; this was followed by a low-dose unenhanced CT, performed for anatomical mapping and attenuation correction. PET attenuation-corrected images were reconstructed using 2 iterations and 21 subsets, with time of flight and corrections for point spread function, attenuation, and scatter. Images were reconstructed onto a 440 × 440 matrix and filtered with a 2.5 mm Gaussian kernel, using the scanner’s software (Syngo VG80). The bilateral temporal, maxillary, occipital, vertebral, and carotid arteries were visually evaluated in a dichotomous manner, as described elsewhere [13].

### 2.3. MRI Acquisition and Analysis

The MRI scans were performed on a Philips 3T Achieva MRI scanner (Philips Medical Systems, Best, The Netherlands) using a 32-channel phased-array head coil. Subjects were scanned with a 3D isotropic variable refocusing flip-angle sequence (VISTA, Philips Medical Systems, Best, The Netherlands). Acquisition used a turbo spin-echo readout scheme (TR 720 ms/TE 24 ms, flip angle 90, SENSE factor 1.3/2 for AP/RL, TSE factor 55 and low–high profile order). Images were acquired over a FOV of 250 mm × 240 mm × 160 mm with isotropic 0.75 mm voxels, and reconstructed to 0.38 mm. Scan time was 8 min.

Three patients in the patient group had the 2-[^18^F]FDG PET/CT and the BB MRI performed on the same date. Concerning the rest of the patient group, the three different imaging modalities were performed on three separate dates, with a maximum of 13 days between the first and last scan, as shown in Table 1. In the control group, the two different imaging modalities were performed on two separate dates.

Two specialists in neuroradiology, masked to all data except sex and birthdate, evaluated the 15 BB MRI scans, separately. If diagnosis disagreement was present, a consensus diagnosis was then made. The same arteries evaluated on 2-[^18^F]FDG PET/CT were evaluated on BB MRI in the axial, sagittal, and coronal plane, respectively, to avoid sequence orientation artefacts. The presence of a hyperintense signal in/along the vessel wall was classified as inflammation and indicative of GCA. In the absence of a hyperintense signal, the BB MRI scan was classified as negative for GCA. Furthermore, an evaluation of the quality of the BB MRI was performed.

Statistical analysis was performed using IBM SPSS Statistics version 25 (IBM, Armonk, NY, USA). Sensitivity, specificity, positive predictive value (PPV), and negative predictive value (NPV) was calculated for BB MRI in diagnosing GCA, 2-[^18^F]FDG PET/CT being the reference test. Median age, CRP, ESR, and time from initiation of high-dose steroid treatment to imaging procedure, respectively, was calculated for patients and control subjects, where relevant. The Mann–Whitney U test was used to test for differences in age between the patient group and the control group. Further statistical analysis was not applicable, due to the small sample size of this exploratory study.

## 3. Results

### 3.1. Participants

A total of ten patients (six females and four males) suspected of having GCA, and five control subjects (two females and three males) were included. The median age of the patients suspected of having GCA was 79.9 (IQR 70.7–85.0) years, and the median age of the control group was 57.5 (IQR 52.6–62.7) years. Baseline characteristics of controls and the patients suspected of having GCA, including fulfillment of American College of Rheumatology (ACR) 1990 GCA classification criteria, are shown in Table 2.

### 3.2. Findings on BB MRI, US, 2-[^18^F]FDG PET/CT, TAB, and Final Diagnosis

The positive and negative diagnoses of GCA on BB MRI, US, 2-[^18^F]FDG PET/CT and TAB, including the final diagnosis of GCA, are shown in Table 3.

**Figure 1:**
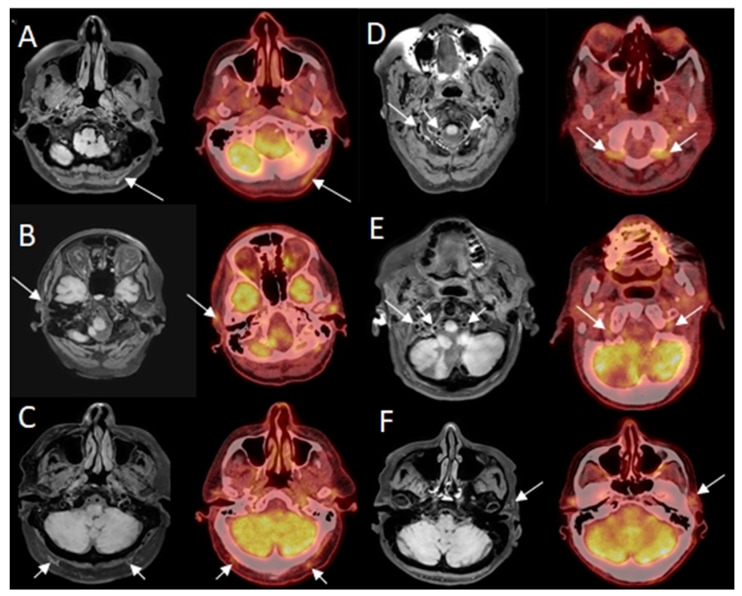
Examples of findings of giant cell arteritis (GCA) on high-resolution T1-weighted 3D black blood without contrast enhancement Magnetic Resonance Imaging (BB MRI) compared to 2-deoxy-2-[^18^F]fluoro-D-glucose positron emission tomography/low-dose computed tomography (2-[^18^F]FDG PET/CT). Hyperintense signal in/related to the artery wall on axial BB MRI (arrows) and increased FDG uptake in arteries on axial fused PET/CT (arrows). Note: BB MRI and 2-[^18^F]FDG PET/CT images are not perfectly aligned, as best visualization of the same affected artery is dependent on head position. Not all affected arteries are marked on 2-[^18^F]FDG PET/CT. (**A**) patient with GCA affecting the left occipital artery; (**B**) patient with GCA affecting the right temporal artery; (**C**) patient with GCA affecting the right and left occipital artery; (**D**) patient with GCA affecting the right and left vertebral artery; (**E**) patient with GCA affecting the right and left vertebral artery; (**F**) patient with GCA affecting the left temporal artery.

**Figure 2:**
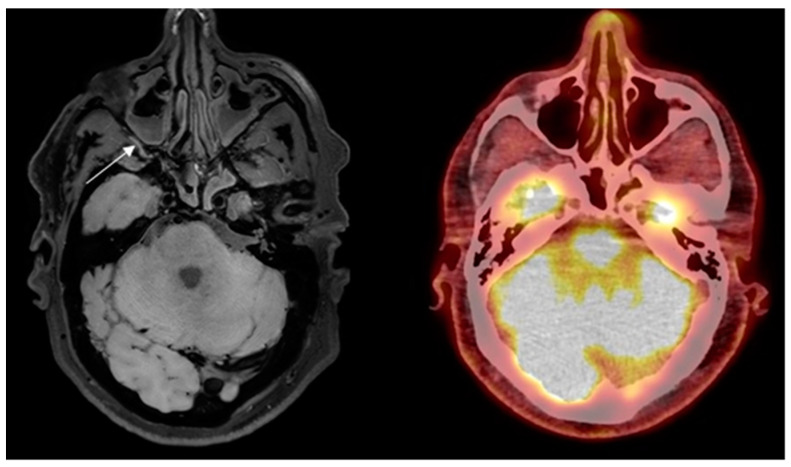
Control subject misinterpreted with GCA in the right maxillary artery on high-resolution T1-weighted 3D black blood without contrast enhancement Magnetic Resonance Imaging (arrow) and no signs of vasculitis on 2-deoxy-2-[^18^F]fluoro-D-glucose positron emission tomography/low- dose computed tomography.

Eight out of ten patients were diagnosed with GCA. Of these eight patients, all eight were correctly identified as positive, independently, by both neuroradiologists reading the BB-MRI images. It was not possible to discriminate whether the inflammation was localized in the periadventitial tissue or in the adventitia. These patients were also correctly diagnosed from 2-[^18^F]FDG PET/CT and US examinations. Regarding the TAB results of the eight GCA-positive patients, one was a false negative, one renounced having the TAB performed, and the remaining six were positive. The patient whose TAB was deemed false negative had the final diagnosis of GCA based on clinical examination after six months, with results including fulfillment of three out of five ACR 1990 GCA classification criteria, elevated CRP, positive US, and positive 2-[^18^F]FDG PET/CT. No ophthalmological consequences of GCA were revealed in the patient by the neuroophthalmological examination. Two out of ten patients were diagnosed as GCA-negative, and were correctly identified as GCA negative by both neuroradiologists reading the BB-MRI images. Both patients were confirmed to be GCA negative by 2-[^18^F]FDG PET/CT, US, and TAB. There were no false negative or false positive 2-[^18^F]FDG PET/CT or US scans when using the final diagnosis after six months as the reference test. Four out of five participants in the control group were negative on the BB MRI, with one false positive. The 2-[^18^F]FDG PET/CT was GCA-negative, as per inclusion criterion. Compared with 2-[^18^F]FDG PET/CT as the reference test, BB MRI had a sensitivity, specificity, PPV, and NPV of 100.0% (95% CI: 63.1–100.0%), 85.7% (95% CI: 42.1–99.6%), 88.9% (95% CI: 68.4–100.0%), and 100.0% (95% CI: 100.0–100.0%), respectively, for diagnosing GCA. Due to motion artefacts, two BB MRI scans were of a poorer quality. This did not affect the diagnostic accuracy of BB MRI compared to the other imaging modalities or TAB. Hence, all 15 BB MRI scans were diagnostic. A head-to-head comparison of affected arteries on US, 2-[^18^F]FDG PET/CT and BB MRI showed some discrepancy. Generally, more arteries were evaluated as inflamed on US and 2-[^18^F]FDG PET/CT than on BB MRI (data not shown). In 14 of the 15 BB MRI scans, the two neuroradiologists made the same diagnosis. In the last case, a consensus diagnosis was made, favoring GCA, which was correct. Examples of imaging results of 2-[^18^F]FDG PET/CT and BB MRI are shown in Figure 1 and Figure 2.

Figure 1 Cranial giant cell arteritis visualized on BB MRI and 2-[^18^F]FDG PET/CT.

Figure 2 False-positive finding of GCA on BB MRI.

The median time from initiation of high-dose steroid treatment in the patient group to performance of US, 2-[^18^F]FDG PET/CT, BB MRI, and TAB was 3.5 (IQR 2.0–4.8), 6.5 (IQR 4.3–12.0), 11.0 (IQR 6.0–14.0), and 9.0 (IQR 7.0–12.3) days, respectively.

In four out of nine patients, the BB MRI was performed before the TAB. Two of the four patients were correctly classified as GCA-negative on BB MRI. The median time from TAB to BB MRI for the remaining five patients was 3.0 (IQR 2.0–5.0) days.

The patient group was significantly older than the control group (*p* < 0.05).

## 4. Discussion

To our knowledge, this is the first study to perform 3D high-resolution T1-weighted BB MRI without contrast enhancement to evaluate cranial vasculitis in GCA patients, compared to controls. Our data shows that BB MRI was accurate in both, correctly establishing and rejecting the GCA diagnosis, with a sensitivity of 100%, a specificity of 86%, a PPV of 89%, and a NPV of 100%, where 2-[^18^F]FDG PET/CT was the reference standard. Furthermore, the diagnostic agreement was high. This indicates that BB MRI could have a role in the diagnostic management of GCA.

The intension of conventional vascular MRI is to visualize the luminal morphology of a vessel. When adding BB MRI with contrast technique, the morphology of the vessel wall is visualized, and thereby adds important information of the pathophysiology of the luminal changes [14]. Previous studies have shown that BB MRI with contrast administration can visualize arterial wall inflammation and periadventitial edema in intracranial vasculopathies, including vasculitis [15,16]. The reported sensitivity and specificity for BB MRI with contrast in diagnosing GCA is 80–91% and 100%, respectively [10,17]. Our study indicates that the characteristic concentric, uniform, and elongated hyperintense signal in/related to the artery wall in GCA could be sufficiently identified on the BB MRI without the use of contrast. It was not possible to pinpoint whether the hyperintense signal was located in the artery wall or the periadventitial space. As histopathological findings in GCA are inflammation in the media and intima, most frequently with giant cells localized in the internal elastic lamina of the arterial wall, and periadventitial/adventitial inflammation is present in most cases, we believe our findings on BB MRI are consistent with GCA [18].

Historically, TAB has been the standard reference in GCA. TAB has its shortcomings, due to it being an invasive procedure with possible false-negative results in segmental affection of the artery and affection of arteries other than the temporal [19]. Non-invasive imaging modalities have now become central tools in supporting the clinical suspicion of GCA. The recently published updated recommendations for the use of imaging in primary large-vessel vasculitis by EULAR stated that the US of the temporal and axillary arteries is the first-line imaging test in GCA, because of a high diagnostic accuracy. Using the clinical diagnosis as the reference standard, the pooled sensitivity and specificity for US in diagnosing GCA are reported to be 88% and 96%, respectively [5]. As in previous 2018 EULAR guidelines, it is still recommended that, in a clear clinical case of cranial GCA and with positive findings on US to support the diagnosis, no further diagnostic workup, including TAB, is necessary [5,20]. As newer PET scanners with higher spatial resolution and sensitivity are now available, studies have been conducted like MRI with contrast; 2-[^18^F]FDG PET/CT has proven accurate in the diagnosis of cranial GCA, which is why both imaging modalities are now recommended as alternatives to US in the diagnostic work-up for cranial GCA [5,13,21,22,23,24,25]. The reported pooled sensitivity and specificity for MRI with contrast in diagnosing cranial GCA, using the clinical diagnosis as the standard reference, were 81% and 96%, respectively. Regarding 2-[^18^F]FDG PET/CT, the reported sensitivity and specificity were 76% and 96%, respectively [26].

Every imaging modality requires experienced performers and readers, and has its benefits and disadvantages. US is widely available, non-invasive, and inexpensive, compared to 2-[^18^F]FDG PET/CT and MRI; it is contrast agent- and radiation-free, and yields a rapid result. US plays a significant role in the fast-track GCA referral-pathway setting, and has reduced visual-impairment complications [27]. Its limitations are mainly the impairment of the visualizing of structures located deeply in the tissue, and structures behind bone, i.e., the vertebral arteries. Special US probes for deeper structures are available on the market. The advantages of MRI are high-resolution images, no radiation exposure, cost-effectiveness, and wide availability. MRI is also a diverse diagnostic tool, where varied and complementary information can be obtained from different scan sequences within one scanning session. Contraindications to MRI are allergy to the MRI contrast agent, severe renal impairment, ferromagnetic implants, and severe claustrophobia. The overall benefit of 2-[^18^F]FDG PET/CT is the full coverage of head to mid-thigh, exposing any involvement of the aorta and larger artery branches. Involvement of the aorta and larger arteries predisposes higher rates of relapse and higher cumulative steroid doses [28]. Furthermore, potential differential diagnosis like other systemic inflammatory diseases, infections, or malignancy, mimicking symptoms of GCA, can be diagnosed. Radiation exposure, long patient-preparation time, costs, the availability only at daytime, and the fact that it should ideally be performed ≤ 3 days of high-dose steroid treatment to ensure a high sensitivity, are its limitations [29]. High-dose steroid treatment can also affect blood glucose levels in patients with diabetes, and thereby decrease the signal-to-noise ratio.

Although the median time from initiation of high-dose steroid and performing the 2-[^18^F]FDG PET/CT was 6.5 (IQR 4.3–12.0) days in this study, no GCA diagnosis was missed, compared to US and the final clinical diagnosis. This could indicate that the advised maximum of three days of steroid treatment before performing the 2-[^18^F]FDG PET/CT can be extended within reasonable limits. If reproducible, this could have a positive impact on the role of 2-[^18^F]FDG PET/CT in the diagnostic management of GCA. The median time before performing the BB MRI was 11.0 (IQR 6.0–14.0) days. This indicates that morphological changes in vasculitis seen on the BB MRI are persistent, and can be visualized even during steroid treatment. Studies have shown that morphological findings of GCA on MRI with contrast are reduced after two weeks of steroid treatment, and suggest that the imaging test should preferably be performed within five days to a week of steroid treatment initiation [24,25]. After initiation of the steroid, the halo sign is reported to persist for at least two weeks, although the size of the halo diminishes significantly during the first week [30]. The infiltrate of abnormal cells in the TAB are present for at least four weeks, and probably even after months after initiation of the steroid [27,31].

The potential benefits of BB MRI without contrast in visualizing vessel inflammation in GCA are high-resolution images, full coverage of cranial arteries, availability, short scan time (8 min), no radiation, and no administration of a contrast agent. Compared to US, the vertebral arteries are easily identified and well visualized. Furthermore, it can be performed in conjuncture with PET, using PET/MRI, with the potential of increasing the diagnostic yield by using two different imaging modalities. Additionally, the patient would benefit from having fewer appointments at the hospital. Limitations in this study are inhomogeneity in age between the control subjects and patients and the small sample size. For this reason, the reported sensitivity and specificity for BB MRI in diagnosing GCA are only indicative, and this explains the large confidence intervals. Preferably, all the BB MRI should have been performed before the TAB. As more arteries were inflamed, the unilateral TAB did not hinder the correct diagnosis from being made in GCA-positive patients. This also holds true for 2-[^18^F]FDG PET/CT. Furthermore, morphological changes after biopsy, i.e., subcutaneous air, can be visualized on low-dose CT, and the increased FDG-uptake due to inflammation after the biopsy can therefore be distinguished from vasculitis. The two (correct) GCA-negative patients on the BB MRI had their scan performed before the TAB. Further studies on possible changes on the BB MRI after biopsy are required. Subjects in the control group were not examined with US, to ensure that they did not have any vascular disorder, although no increased FDG-uptake indicating arteriosclerosis was visualized on 2-[^18^F]FDG PET/CT. In only one case did the findings from 2-[^18^F]FDG PET/CT and BB MRI differ. On the BB MRI, a hyperintense signal was observed in/related to the right maxillary artery wall, suggestive of GCA. This signal artifact can be due to slow flowing of the blood, as the suppression of the signal is flow-dependent [8,32]. We do acknowledge that there are other potential pitfalls producing a hyperintense signal in/related to the vessel wall, i.e., a thrombus with recanalization in the temporal artery which requires a TAB to exclude the GCA diagnosis. Care should therefore be taken not to misinterpret hyperintense signals due to incomplete blood signal suppression as inflammation in GCA.

In conclusion, this explorative study indicates that BB MRI can accurately establish or reject the GCA diagnosis. As the information it provides differs from using contrast accumulation, it can be a valuable supplement, as an alternative imaging test with a short scan time, in the investigation of cranial GCA in patients, or even a substitute in patients with severe renal impairment or contrast allergy. Based on these results, further investigation of the specificity and sensitivity of BB MRI for GCA on a larger scale is warranted.

## Figures and Tables

**Table 1:** Days between performance of the different imaging modalities in the patient group.

Patient	US	2-[^18^F]FDG PET/CT	BB MRI
1	0	1	1
2	0	7	12
3	0	9	13
4	0	1	3
5	0	1	7
6	1	0	0
7	0	3	3
8	0	6	9
9	0	1	11
10	0	6	0

For each patient day, 0 indicates when the first imaging modality is performed and the following numbers of days indicate time to performance of the two remaining imaging modalities from day 0. Abbreviations: US = vascular ultrasound; 2-[^18^F]FDG PET/CT = 2-deoxy-2-[^18^F]fluoro-D-glucose positron emission tomography/low-dose computed tomography; BB MRI = High-resolution T1-weighted 3D black blood without contrast enhancement Magnetic Resonance Imaging.

**Table 2:** Patient and control characteristics.

	Patients	Controls
Sex, *n* (%)		
Male	4 (40)	3 (60)
Female	6 (60)	2 (40)
Age, median (IQR)	79.9 (70.7–85.0)	57.5 (52.6–62.7) (*p* < 0.005)
CRP, median (IQR)	55.5 (21.3–120.0)	n/a
ESR, median (IQR)	65.0 (41.3–83.5)	n/a
ACR criteria, *n* (%)		
≥3	9 (90)	n/a
<3	1 (10)	n/a

Abbreviations: IQR = Inter Quartile Range; CRP = C-reactive Protein; ESR = Erythrocyte Sedimentation Rate; ACR criteria = The American College of Rheumatology (1990) giant-cell-arteritis classification criteria: (1) Age at onset ≥ 50 years; (2) Headache as a new symptom; (3) Temporal artery abnormality such as tenderness to palpation or decreased pulsation; (4) ESR ≥ 50 mm; (5) Abnormal temporal biopsy, with features characteristic of giant cell arteritis. n/a = not applicable.

**Table 3:** Diagnosis of GCA on US, 2-[^18^F]FDG PET/CT, BB MRI, TAB, and final diagnosis.

	US	2-[^18^F]FDG PET/CT	BB MRI	TAB	Final Diagnosis
Patient 1	Positive	Positive	Positive	Positive	GCA
Patient 2	Positive	Positive	Positive	Positive	GCA
Patient 3	Positive	Positive	Positive	Positive	GCA
Patient 4	Positive	Positive	Positive	Negative	GCA
Patient 5	Positive	Positive	Positive	Not performed	GCA
Patient 8	Positive	Positive	Positive	Positive	GCA
Patient 9	Positive	Positive	Positive	Positive	GCA
Patient 10	Positive	Positive	Positive	Positive	GCA
Patient 6	Negative	Negative	Negative	Negative	Not GCA
Patient 7	Negative	Negative	Negative	Negative	Not GCA
Control 1	n/a	Negative	Negative	n/a	n/a
Control 2	n/a	Negative	Negative	n/a	n/a
Control 3	n/a	Negative	Positive	n/a	n/a
Control 4	n/a	Negative	Negative	n/a	n/a
Control 5	n/a	Negative	Negative	n/a	n/a

Abbreviations: GCA = giant cell arteritis; US = vascular ultrasound; 2-[^18^F]FDG PET/CT = 2-deoxy-2-[^18^F]fluoro-D-glucose positron emission tomography/low-dose computed tomography; BB MRI = High-resolution T1-weighted 3D black blood without contrast-enhancement Magnetic Resonance Imaging; TAB = temporal artery biopsy; Final diagnosis = clinical evaluation after six months, including findings on US, 2-[^18^F]FDG PET/CT, and TAB; n/a = not applicable.

## Data Availability

The data presented in this study are available on request from the corresponding author. The data are not publicly available do to privacy restrictions.
